# Al_2_O_3_-Modified Polymer-Derived Ceramic SiCN High-Temperature Anti-Oxidative Composite Coating Fabricated by Direct Writing

**DOI:** 10.3390/polym14163281

**Published:** 2022-08-12

**Authors:** Chao Wu, Xiaochuan Pan, Fan Lin, Guochun Chen, Lida Xu, Yingjun Zeng, Yingping He, Daoheng Sun, Zhenyin Hai

**Affiliations:** Department of Mechanical and Electrical Engineering, School of Aerospace Engineering, Xiamen University, Xiamen 361005, China

**Keywords:** polymer-derived ceramics, anti-oxidative coating, high-temperature stability, thin film sensor

## Abstract

A reliable protective layer is one of the main challenges in preventing oxidation of thin film sensors to achieve accurate, effective, and stable readings at high temperatures. In this work, an Al_2_O_3_-modified polymer-derived ceramic SiCN composite coating fabricated by a direct-writing technique is utilized as a protective layer for thin film sensors. The microstructure evolution of the Al_2_O_3_/SiCN films is examined herein. The protective layer exhibits excellent oxidation resistance and thermal stability at high temperatures up to 1000 °C, which contributes to improving the stability and lifetime of thin film sensors in extreme environments. The TiB_2_/SiCN thin film resistive grid with the Al_2_O_3_/SiCN composite film as a protective layer is fabricated and tested. The results indicate that the coating can protect the TiB_2_/SiCN thin film resistive grid at high temperatures up to 1000 °C, which is about 200 °C higher than that of the TiB_2_/SiCN thin film resistive grid without a protective layer. The resistance change rates of the TiB_2_/SiCN thin film resistive grid with a protective layer are 0.5%/h at 900 °C and 10.7%/h at 1000 °C.

## 1. Introduction

Thin film sensors (TFSs) for high temperatures, such as high-temperature thin film strain gauges and heat-flow meters, are widely used in aerospace and aircraft industries, coal gasification systems, material processing systems, and other fields owing to their high sensitivity, ease of integration, fast response, and easy-to-extract signals [1,2,3,4,5,6]. Different from ordinary TFSs, thermal stability at high temperatures is the first design principle of high-temperature TFSs [2,7,8]. When TFSs work in a high-temperature environment for a long time, their sensitive materials are often severely oxidized, resulting in degradation of the performance and reliability of the sensor [9,10,11]. For example, Babak B. et al. proved that TiB_2_ film has a higher melting point, but its resistance to high-temperature oxidation is poor. Its composition changes in air at 500 °C, making it difficult to use in high-temperature aerobic environments [12]. Therefore, anti-oxidation protective layers or oxygen barrier layers are crucial to preventing the oxidation of sensitive materials and improving the high-temperature stability and reproducibility of TFSs.

Materials used for high-temperature TFS protective layers should have high melting points, thermal stabilities, and anti-oxidation capacities at the same time. Ultra-high-temperature ceramics (UHTCs) and oxides are widely used as high-temperature oxidation-resistance materials [13]. UHTCs including the borides, carbides, and nitrides of transition metals are endowed with ultra-high melting points, excellent mechanical properties, and ablation resistance at elevated temperatures. Carbide-based UHTCs are easier to oxidize at low temperatures in aerobic environments. Compared with carbides, transition-metal diborides are characterized by good oxidation resistance owing to in situ dense B_2_O_3_ glasses on the surface. However, both carbides (electrical resistivity: 30–109 μΩ·cm) and transition-metal diborides (electrical resistivity: 10–30 μΩ·cm) are highly conductive and cannot be directly deposited on the surface of TFSs, limiting their application for protective layers to some extent [13]. Al_2_O_3_ is generally used as an anti-oxidation material due to its high electrical resistance, thermal stability, and low oxygen diffusion coefficient [2]. Heterolayer Al_2_O_3_-ZrO_2_/Al_2_O_3_ protective coatings fabricated by electron beam evaporation enabled a PdCr-sensitive thin film to operate stably at 800 °C [7]. An Al_2_O_3_/Al bilayer film with Al as the sacrificial layer can significantly improve the oxidation resistance of the substrate or sensitive film at 750–800 °C [14,15].

Polymer-derived ceramics (PDCs), which have gained a reputation as promising high-temperature materials due to their thermal stability and corrosion/oxidation resistance in harsh environments, have been used as environmental barrier coatings for metallic materials [16,17]. The properties and composition of PDC coatings can be changed by tailoring the chemical structure of the precursors or by adding fillers [18,19]. Composite coatings have been developed as oxidation and carburization barriers on steel using a precursor matrix, and TiSi_2_ or ZrSi_2_ as reactive fillers. After cyclic oxidation testing of the coated samples at 800 °C, the coating system remained undamaged and no oxidation occurred on the steel substrate, demonstrating the effectiveness of the PDC anti-oxidative coating [20,21]. However, most PDC coatings for the thermal protection of metals are not electrically insulating, and it is uncertain whether they generate electrical shunting and affect the performance of TFSs. For TFSs, anti-oxidative coatings with high electrical insulation resistance are required. In this study, an Al_2_O_3_-modified PDC-SiCN high-temperature anti-oxidation composite coating fabricated by a direct ink writing (DIW) technique was developed. The PDC-SiCN pyrolyzed at 800 °C is insulating (the DC conductivity at room temperature is in the range of 10^−14^–10^−12^ S/cm) [22]. This protective layer is suitable for applications up to 1000 °C. The microstructure and composition of the protective layer were investigated in detail. The high-temperature stability of TiB_2_/SiCN thin film resistive grids with an Al_2_O_3_/SiCN film as the protective coating was tested.

## 2. Materials and Methods

**Figure 1:**
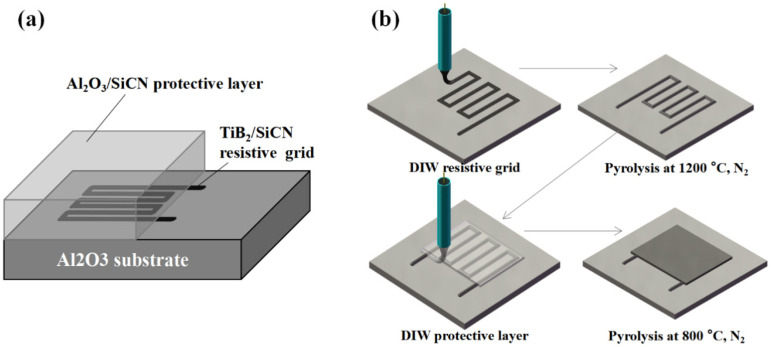
Schematic illustration of (**a**) structure and (**b**) process.

Fabrication. The film structure and process are shown in Figure 1. TiB_2_/SiCN thin film resistive grids and the Al_2_O_3_/SiCN protective layer were fabricated by the DIW technique. TiB_2_/SiCN thin film resistive grids were fabricated using commercially available PSN2 (Chinese Academy of Sciences, Beijing, China) filled with TiB_2_ nanopowders (average diameter: 50 nm, Shanghai Chaowei NanoTechnology, Shanghai, China) as liquid precursors. The viscosity of the PSN2 was 50–80 cP at room temperature, which is beneficial to improving the wettability of nanoparticles (NPs) and substrates. The printed resistive grids were pyrolyzed at 1200 °C in a N_2_ atmosphere to form functional ceramic films with electrical conductivity. PSN2 filled with Al_2_O_3_ nanopowder (diameter: 100–200 nm, Zhongye New Materials, Changsha, China) was utilized as a liquid precursor for the protective layer. The amount of Al_2_O_3_ nanopowder used was 10 wt%, 20 wt%, 30 wt%, and 40 wt%. The printed films were pyrolyzed at 800 °C in N_2_ atmosphere to form an Al_2_O_3_/SiCN composite film. The ceramic films were denoted as Al_2_O_3_ (10 wt%)/SiCN, Al_2_O_3_ (20 wt%)/SiCN, Al_2_O_3_ (30 wt%)/SiCN, and Al_2_O_3_ (40 wt%)/SiCN.

Characterization. Scanning electron microscopy (SEM, SUPRA55 SAPPHIRE, Shanghai, China) coupled with energy-dispersive spectroscopy (EDS) was used to characterize the morphology of the samples obtained. X-ray photoelectron spectroscopy (XPS; Thermo Scientific ESCALAB Xi+) was performed to determine the chemical bonds. An atomic force microscope (Oxford Instruments Asylum Research Cypher ES, Oxfordshire, UK) was used to characterize the morphology of the samples obtained. The XRD pattern was acquired using an X-ray diffractometer (XRD-7000, Shimadzu Corp., Kyoto, Japan). A home-made temperature–resistance testing system, which consisted of a tube furnace, a K-type thermocouple, a data acquisition device, and a computer, was used to test the temperature–resistance characteristics and high-temperature electrical stability of the solder joints.

## 3. Results

### 3.1. Film Morphology

**Figure 2:**
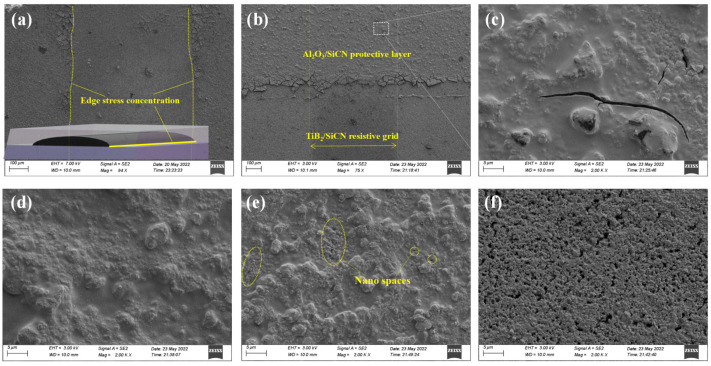
SEM images of (**a**) SiCN, (**b**,**c**) Al_2_O_3_ (10 wt%)/SiCN, (**d**) Al_2_O_3_ (20 wt%)/SiCN, (**e**) Al_2_O_3_ (30 wt%)/SiCN, and (**f**) Al_2_O_3_ (40 wt%)/SiCN.

**Figure 3:**
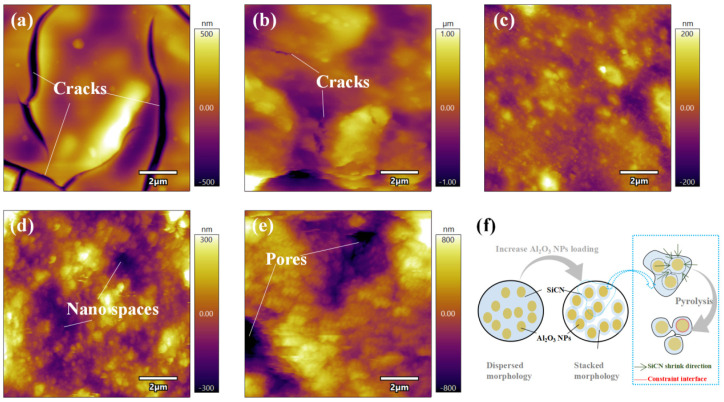
AFM images of (**a**) SiCN, (**b**) Al_2_O_3_ (10 wt%)/SiCN, (**c**) Al_2_O_3_ (20 wt%)/SiCN, (**d**) Al_2_O_3_ (30 wt%)/SiCN, and (**e**) Al_2_O_3_ (40 wt%)/SiCN. (**f**) Schematic illustration of the microstructure evolution of the Al_2_O_3_/SiCN film with increasing Al_2_O_3_ NP loading.

SEM images of the samples are presented in Figure 2. It can be clearly seen that there were many cracks and exfoliation in the SiCN film at the edge of the resistive grid. A polymer-to-ceramic conversion is typically associated with a mass loss in the range of 10–30% and a size shrinkage of 40–70% [18]. This results in the critical thickness of PDC-SiCN films typically being less than 3 μm [23]. Moreover, the edges of films and the abrupt shape changes of heterointerfaces are considered to be stress concentrations [24,25]. The stress at these locations is often much greater than the average stress across the film, resulting in cracks being generated at the stress concentration locations and further propagating [26]. The SEM images of Al_2_O_3_ (10 wt%)/SiCN film are shown in Figure 2b,c. Compared with the SiCN film, although there were tiny cracks on the surface of the Al_2_O_3_ (10 wt%)/SiCN film, the cracks at edges were obviously eliminated. Figure 2d shows the microstructure of an Al_2_O_3_ (20 wt%)/SiCN film. The composite film was dense without cracks or pores. Figure 2e shows the presence of nano spaces in the Al_2_O_3_ (30 wt%)/SiCN film. Further increasing the Al_2_O_3_ loading, these interconnected micro-porosities or micro-channels were more obvious (Figure 3f).

For a quantitative roughness comparison, the films were characterized by AFM, and the results are shown in Figure 3. The root mean square (RMS) surface roughness calculated by AFM was found to be 143.65 nm, 250.11 nm, 35.61 nm, 86.35 nm, and 264.35 nm for the films SiCN, Al_2_O_3_ (10 wt%)/SiCN, Al_2_O_3_ (20 wt%)/SiCN, Al_2_O_3_ (30 wt%)/SiCN, and Al_2_O_3_ (40 wt%)/SiCN, respectively. The roughness first decreased and then increased with the increase in Al_2_O_3_ filler weight percent. With the increase in Al_2_O_3_ loading, the morphology of the film underwent significant changes, gradually transforming from microcracked to dense and then to porous. It is worth noting that PSN2 has a lower viscosity and good wetting property. Additionally, the interaction between polysilazane and the oxygen/hydroxyl group (-OH) adsorbed on the surface of Al_2_O_3_ NPs could contribute to increasing the dipole–dipole interaction or hydrogen bond between Al_2_O_3_ and polysilazane residual polar groups such as hydroxyl and amine groups [27]. Therefore, the surface of Al_2_O_3_ NPs is easily wrapped with a layer of polysilazane solution, and after high-temperature pyrolysis, the core–shell structure of SiCN@Al_2_O_3_ is formed. This contributes to increasing the performance of the protective layer. Pores between particles can provide fast channels for high-temperature oxygen diffusion. When the Al_2_O_3_ loading is low, the excess PSN2 ceramizes and forms a structure in which Al_2_O_3_ NPs are dispersed in the SiCN glassy matrix. As the Al_2_O_3_ loading increases, the microstructure gradually evolves from the dispersed form of Al_2_O_3_ to the stacked form of Al_2_O_3_ particles coated with SiCN (Figure 3f). The difference in morphology is because Al_2_O_3_ NPs act as the skeleton in the composite film under high Al_2_O_3_ loading, which limits free shrinkage of the SiCN phase during heat treatment. In fact, the SiCN phase in the whole system uniformly undergoes volume shrinkage during the pyrolysis process. The presence of Al_2_O_3_ particles increases the interface volume ratio of Al_2_O_3_/SiCN. The adhesion between the Al_2_O_3_/SiCN interfaces acts as a barrier against the shrinkage force, which allows the SiCN to undergo in-plane tensile stress along the Al_2_O_3_/SiCN interface, resulting in shrinkage of the SiCN film along the normal direction of the Al_2_O_3_/SiCN interface. The final microstructure of the film inherits the Al_2_O_3_ framework structure and evolves into a porous structure.

**Figure 4:**
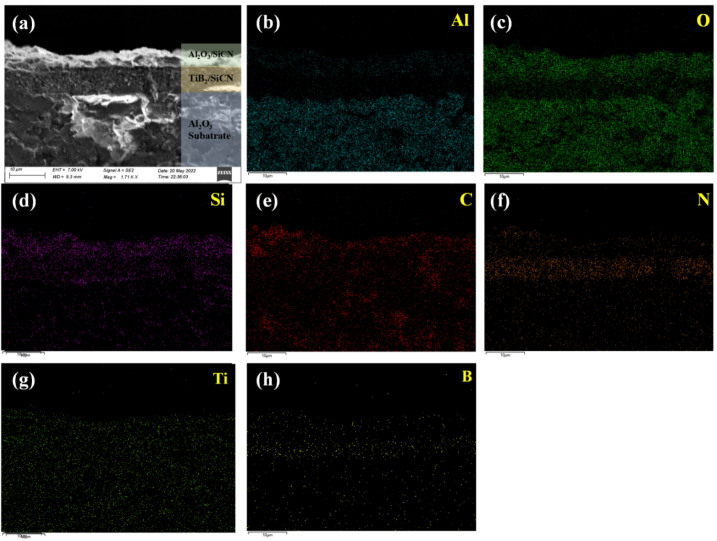
The cross-sectional SEM-EDS images of Al_2_O_3_/SiCN-TiB_2_/SiCN film on Al_2_O_3_ substrate after oxidation at 1000 °C for 1 h: (**a**) SEM, (**b**) Al element map, (**c**) O element map, (**d**) Si element map, (**e**) C element map, (**f**) N element map, (**g**) Ti element map, and (**h**) B element map.

Although the oxidation resistance of Al_2_O_3_ is excellent, high operating temperatures increase the rate of degradation, such as time-dependent deformation (creep), resulting in the loss of structural integrity and, ultimately, failure [9,28]. Owing to the brittleness of Al_2_O_3_, micro-cracks easily form, resulting in a decrease in the oxidation resistance of the Al_2_O_3_ protective layer [10,29]. The SEM cross-sectional image of the Al_2_O_3_ (20 wt%)/SiCN film after oxidation at 1000 °C for 1 h in air is presented in Figure 4a. All two layers are dense and pinhole-free, with a visible interface. Layers are tightly bonded to each other. No porous framework structure normally associated with B_2_O_3_ evaporation at high temperature is observed on the surface or cross section of Al_2_O_3_ (20 wt%)/SiCN films [30]. The element mappings of aluminum, oxygen, silicon, carbon, nitrogen, titanium, and boron were identified by EDS, as shown in Figure 4b–h. It should be noted that the oxygen content is much lower in the TiB_2_/SiCN sensitive layer. This indicates that the Al_2_O_3_/SiCN film has good thermal stability and oxidation resistance.

### 3.2. Film Composition

**Figure 5:**
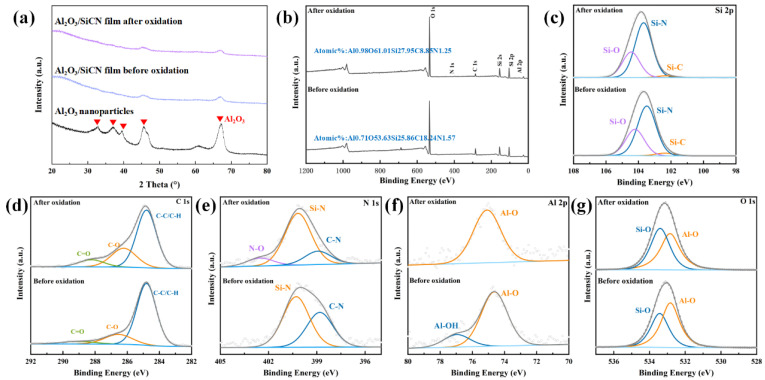
(**a**) XRD patterns of Al_2_O_3_ filler and Al_2_O_3_/SiCN film. XPS spectra of the as-prepared Al_2_O_3_(20 wt%)/SiCN film: (**b**) XPS spectra, (**c**) Si 2p, (**d**) C 1s, (**e**) N 1s, (**f**) Al 2p, and (**g**) O 1s.

The XRD patterns of the Al_2_O_3_/SiCN film before and after oxidation at 1000 °C for 1 h are presented in Figure 5a. Compared with Al_2_O_3_ fillers, the peak intensity of Al_2_O_3_/SiCN was reduced significantly, which is related to the SiCN coated on the surface of Al_2_O_3_ particles. Moreover, no diffraction peaks of SiCN-related crystals were detected, indicating that PDC-SiCN mainly exists in the form of glass. After 1 h of oxidation at 1000 °C, no new phase was found, indicating the thermal stability of the film. A depth analysis of XPS was used to characterize the chemical bond of the Al_2_O_3_/SiCN film before and after oxidation at 1000 °C. Figure 5b shows wide-scan spectra of Al_2_O_3_/SiCN films to identify the surface element present with quantitative analysis. The elements present in these samples are mainly aluminum, oxygen, silicon, carbon, and nitrogen. Because the surface of Al_2_O_3_ particles was covered with a layer of dense SiCN, the aluminum content in the films measured before and after oxidation was very low, 0.71 and 0.91 atomic%, respectively. After oxidation at 1000 °C for 1 h, the contents of carbon and oxygen in the protective layer changed significantly. Carbon content decreased from 18.24 atomic% to 8.85 atomic%, while oxygen content increased from 53.63 atomic% to 61.01 atomic%. To investigate the chemical bonding type of the Al_2_O_3_/SiCN film, the XPS spectra were fit by the Gaussian–Lorentzian function (Figure 5c–g). The main chemical bonds including Si-N, Si-C, Si-O, C-C/H, and C-N illustrated the generation of the Si-C-N-O network structure [31]. Some studies have shown that Al_2_O_3_/SiCN or Al_2_O_3_/SiC composite films prepared by sputtering generate new crystal phases, such as Al_2_SiO_5_ and Al, after experiencing high temperatures over 1000 ℃ [9]. There are only Al-O bonds in the Al 2P spectra before and after oxidation, but no Al-Al, Al-Si, or Al-C bonds.

In summary, the Al_2_O_3_/SiCN film as a protective layer has two remarkable characteristics: excellent thermal stability and a unique amorphous wrapping and filling structure. Unlike the physical vapor deposition, electron beam evaporation, and other thin film deposition processes, the precursor conversion method not only improves interlayer adhesion but also acts as a barrier to oxygen diffusion. The formation of the PDC-SiCN amorphous phase not only fills the pores between the Al_2_O_3_ NPs but also leads to a reduction in the high-angle grain boundaries, generally fast diffusion channels of oxygen [9]. In this system, Al_2_O_3_ acts as a filler, increasing the critical thickness of the film, and PDC-SiCN acts as a binder, reducing oxygen diffusion. As a result, the oxidation-resistance performance of the composite film was further improved.

### 3.3. Protection Performance

**Figure 6:**
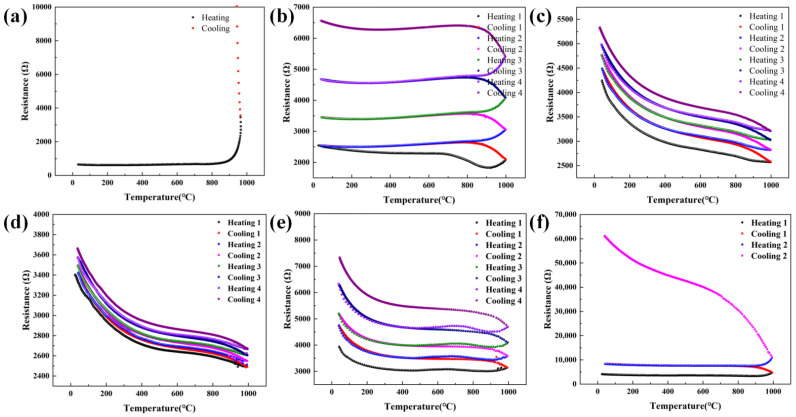
R–T curves of TiB_2_/SiCN resistive grids: (**a**) without protective layer, (**b**) with SiCN protective layer, (**c**) with Al_2_O_3_ (10 wt%)/SiCN protective layer, (**d**) with Al_2_O_3_ (20 wt%)/SiCN protective layer, (**e**) with Al_2_O_3_ (30 wt%)/SiCN protective layer, and (**f**) with Al_2_O_3_ (40 wt%)/SiCN protective layer.

Oxidation resistance of thin film sensors, particularly at high temperatures, is critical for the lifetime and performance of the sensor. The effectiveness of the Al_2_O_3_/SiCN protective layer was evaluated by measuring the resistance-temperature (R-T) curve of the TiB_2_/SiCN resistive grid. The R-T characteristics of TiB_2_/SiCN thin film resistive grids using different protective layers are shown in Figure 6a. At 800–1000 °C, the resistance of the TiB_2_/SiCN resistive grid without a protective layer experienced a rapid increase, attributed to the high-temperature oxidation of the resistive grid and the evaporation of the oxidation product B_2_O_3_ [13]. In contrast, resistive grids with Al_2_O_3_/SiCN protective layers can survive high temperatures up to 1000 °C. After each thermal cycle at 1000 °C, the average resistance deviations of the TiB_2_/SiCN resistive grid were 39%, 6.4%, 1.8%, 17%, and 21%, corresponding to the SiCN, Al_2_O_3_ (10 wt%)/SiCN, Al_2_O_3_ (20 wt%)/SiCN, Al_2_O_3_ (30 wt%)/SiCN, and Al_2_O_3_ (40 wt%)/SiCN protective layers, respectively. Even the porous Al_2_O_3_ (30 wt%)/SiCN protective layer protects the TiB2/SiCN resistive grid, which is inseparable from the effect of PDC-SiCN in the protective layer. Owing to the relatively high density of Al_2_O_3_ (20 wt%)/SiCN film, the Al_2_O_3_ (20 wt%)/SiCN protective layer shows better high-temperature oxidation resistance, which is 200 °C higher than the failure temperature of some other protective layers. These layers include, Al_2_O_3_ and SiO_2_ single/composite film [7,10,11,14,15,32] and are comparable to the Al_2_O_3_/ZrBN-SiCN/Al_2_O_3_ heterogeneous film [15].

**Figure 7:**
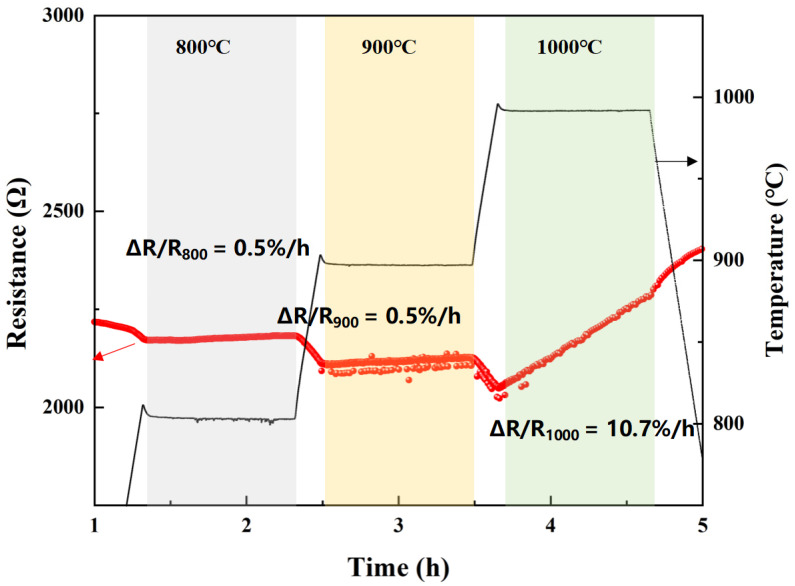
Resistance change curves of TiB_2_/SiCN resistive grid with Al_2_O_3_ (20 wt%)/SiCN protective layer at 800 °C, 900 °C, and 1000 °C for 1 h.

To further determine the influence of the Al_2_O_3_(20 wt%)/SiCN protective layer on the thermal stability of the TiB_2_/SiCN film, the film was kept at 800 °C, 900 °C, and 1000 °C for 1 h in an air atmosphere. The change in resistance with time was measured, as shown in Figure 7. The resistance change rates of the TiB_2_/SiCN resistive grid kept at 800 °C and 900 °C for 1 h were both 0.5%, indicating excellent oxidation resistance of the Al_2_O_3_ (20 wt%)/SiCN film and thermal stability of the whole system. The resistance change rate of the resistance grid kept at 1000 °C for 1 h was 10.7%. Although the resistance of the resistive grid increased slowly, it still maintained excellent electrical conductivity.

## 4. Conclusions

Thermal stability is necessary for high-temperature TFSGs. An Al_2_O_3_-modified PDC-SiCN high-temperature oxidation-resistant composite coating was fabricated by direct writing for high-temperature application. Owing to the dense morphology and excellent thermal stability, the Al_2_O_3_/SiCN composite coating prepared in this study exhibited excellent high-temperature oxidation resistance and protection up to 1000 °C. Under the action of the protective layer, the resistance stability of the TiB_2_/SiCN film has been effectively improved, from complete failure at more than 800 °C to a resistance change rate of 0.5%/h at 900 °C and a resistance change rate of 10.7%/h at 1000 °C. This means a high capability of sensitive films at high temperatures. A protective layer that is effective on thin film thermocouples, thin film strain gauges, and thin film heat flux sensors will be researched in the future.

## Data Availability

The data used to support the fifindings of this study are included within the article.
